# Metabolic patterns in insulin-resistant male hypogonadism

**DOI:** 10.1038/s41419-018-0587-9

**Published:** 2018-04-22

**Authors:** Federica Gevi, Giuseppina Fanelli, Lello Zolla

**Affiliations:** 1 Department of Science and Technology for Agriculture, Forestry, Nature and Energy (DAFNE), University of Tuscia, Viterbo, Italy; 2 Department of Ecological and Biological Sciences (DEB), University of Tuscia, Viterbo, Italy

## Abstract

Male hypogonadism associated with insulin resistance (IR) very often leads to metabolic syndrome, at variance with hypogonadism in its first stadium of insulin sensitivity (IS). A plasma metabolomic investigation of these patients can provide useful information in comparison with the values of IS patients. To this aim plasma from insulin-resistant males with hypogonadism were analysed by using ultra high-performance liquid chromatography (UHPLC) and high-resolution mass spectrometry (HRMS). Thus, metabolites were compared to the controls through multivariate statistical analysis and grouped by metabolic pathways. Metabolite database searches and pathway analyses identified imbalances in 18–20 metabolic pathways. Glucose metabolism (e.g., glycolysis and the Krebs cycle) is fuelled by amino acids degradation, in particular of branched amino acids, in individuals with lean body mass. Gluconeogenesis is strongly activated. Some crucial pathways such as glycerol are skewed. Mitochondrial electron transport is affected with a reduction in ATP production. Beta-oxidation of short and medium chain fatty acids did not represent an energy source in hypogonadism, at variance with long and branched fatty acids, justifying the increase in fat mass. Carnosine and β-alanine are strongly reduced resulting in increased fatigue and mental confusion. A comparison of IR with IS male hypogonadism will contribute to a better understanding of how these two hormones work in synergy or antagonise each other in humans. It could also help to select patients who will respond to hormone treatment, and provide accurate biomarkers to measure the response to treatment eventually leading to better strategies in preventing systemic complications in patients not fit for hormone replacement therapy.

## Introduction

Male hypogonadism is a disorder characterised by low levels of the hormone testosterone, which can arise from various testicular (primary) and central (secondary) causes^1^. It can present symptoms and signs related to the sexual sphere, such as reduced libido, erectile dysfunction, decreased volume of ejaculate, and infertility, as well as involving the whole organism, such as reduced muscle strength, reduced bone strength, anaemia, and low mood (sadness, sense of hopelessness, profound sense of fault, etc.)^2^. Thus, hypogonadism appears to show different symptoms, which are probably induced by different molecular mechanisms^3–7^ that are still unclear. In fact the reversal of these conditions when eugonadism is restored by androgen replacement therapy was observed in some but not all patients. This observation supports the notion that several factors can give rise to similar symptoms and that more than one hormone participates in the process^8^. Among subjects with low testosterone levels (regardless to diabetes), some patients have high and others have low insulin levels, as measured by HOMAi (Homoeostatic Model Assessment for Insulin Resistance-index)^3,9^. Clearly, in these two different groups the inflammatory mediators increase differently and interfere with insulin signalling in different ways. In support of this hypothesis, recent studies^10^ have shown that there is a positive correlation between the testosterone levels and insulin sensitivity. These studies suggested that testosterone plays a crucial role in regulating insulin sensitivity, with low hormone levels increasing the insulin resistance (IR)^10,11^.

Based on the above reported information, we performed two different investigations in patients under very strict conditions, where testosterone levels were low but insulin levels were extremely different. In our opinion, a better understanding of the altered pathways in two different types of hypogonadism may contribute to a better understanding of how these two hormones work in synergy or antagonistically. It is well accepted that the application of metabolomics offers a variety of scientific opportunities^12^ to improve the clinical management of testosterone deficiency in men. Metabolomics, in fact, is a widely used bio-analytical methodology in systems biology since it is able of collecting a large number of features in human plasma. The new findings could help in selecting patients who will respond to hormone treatment, and to provide accurate biomarkers for evaluating the response to treatment. Moreover, a metabolic investigation of plasma from hypogonadic men before and after testosterone treatment could shed light on the molecular mechanisms of this condition, as well as on related biochemical pathways thus accounting for the overall benefit in insulin sensitivity observed in clinical trials.

In the present investigation, we have selected hypogonadic patients showing insulin-resistant HOMAi > 2.5 and other strict conditions as low testosterone concentrations (<8 nmol/L), high insulin (>18), and elevated BMI (Body Mass Index) (30.29 ± 4.43). A comparison between data collected here and those previously performed on insulin-sensitive patients (IS)^13^ is also discussed. The data showed that in these patients, the main glucose metabolic pathways (e.g., glycolysis and the Krebs cycle) are strongly reduced, and both are mainly fuelled by amino acids degradation. Mitochondrial beta-oxidation of free fatty acids was not observed, while increased lipogenesis and gluconeogenesis were induced. Glycerol shuttle, as well as malate decreased significantly. Carnosine and β-alanine were strongly reduced, while betaine, an osmotic pressure regulator, was increased.

## Materials and methods

We evaluated 15 hypogonadal male patients and 15 age-matched and BMI-matched controls. We chose IR hypogonadal patients using very strict conditions as follows: low testosterone concentrations (<8 nmol/L), high insulin (>18), HOMAi > 2.5 and elevated BMI (30.29 ± 4.43) as shown in Table 1. All the enroled subjects were informed regarding the study protocol and gave their written consent. A diagnosis of hypogonadism was based on the presence of clinical symptoms related to this condition (e.g., delayed sexual development, reduced libido or erectile dysfunction) and on the results of standard hormone exams. The participants of the control group were selected from healthy males who were referred to the Outpatient Clinic of Endocrinology and Metabolism for check-up. As shown in Table 1, no differences were found in the baseline characteristics between the groups.

**Table 1:** Characteristics of study participants

TABLE I	Ctrl	IR
Subjects	n-15	n-15
Age (years)	42.6 ± 14.41	49.13 ± 13.5
BMI (Kg/m^2^)	23.94 ± 2.54	30.48 ± 3.011
Testosterone	20.87 ± 7.36	5.53 ± 3.36
Glucose	94 ± 31.05	106.13 ± 24.65
Insuline	7.06 ± 2.10	18.85 ± 6.94
Tg (mmol/l)	87.8 ± 45.21	226 ± 31.2
Cholesterol (mmol/l)	203.4 ± 34.50	235.13 ± 39.16
HDL Cholesterol (mmol/l)	55.8 ± 10.76	42 ± 15.44
LDL Cholesterol (mmol/l)	129.6 ± 32.14	142.75 ± 34.7

Data are presented as the mean ± SD. Statistical differences were determined using Tukey’s multiple comparisons where significant interactions were observed

*BMI* body mass index, *TG* triglycerides, *LDL* low-density lipoprotein, *HDL* high-density lipoprotein

### Plasma collection and metabolite extraction

Metabolites were extracted by adding 200 µl of each plasma sample to 600 µl of a chloroform/methanol/water (1:3:1 ratio) solvent mixture stored at −20 °C. Samples were vortexed for 1 min and left on ice for 2 h for complete protein precipitation. The solutions were then centrifuged for 15 min at 15,000×*g*.

## UHPLC-HRMS

Twenty-microliter of plasma supernatant samples was injected into an ultra high-performance liquid chromatography (UHPLC) system (Ultimate 3000, Thermo) and run on a positive mode: samples were loaded onto a Reprosil C18 column (2.0 mm × 150 mm, 2.5 μm—Dr Maisch, Germany) for metabolite separation. Chromatographic separations were made at a column temperature of 30 °C and a flow rate of 0.2 ml/min. For positive ion mode (+) MS analyses, a 0–100% linear gradient of solvent A (ddH_2_O, 0.1% formic acid) to B (acetonitrile, 0.1% formic acid) was employed over 20 min, returning to 100% A in 2 min and holding solvent A for a 6-min post-time hold. Acetonitrile, formic acid, and HPLC-grade water and standards (≥98% chemical purity) were purchased from Sigma Aldrich. The UHPLC system was coupled online with a Q Exactive mass spectrometer (Thermo) scanning in full MS mode (2 μscans) at a resolution of 70,000 in the 67 to 1000 *m*/*z* range, a target of 1106 ions and a maximum ion injection time (IT) of 35 ms with 3.8 kV spray voltage, 40 sheath gas, and 25 auxiliary gas. The system was operated in positive ion mode. Source ionisation parameters were as follows: spray voltage, 3.8 kV; capillary temperature, 300 °C; and S-Lens level, 45. Calibration was performed before each analysis against positive or negative ion mode calibration mixes (Pierce, Thermo Fisher, Rockford, IL) to ensure error of the intact mass within the sub ppm range. Metabolite assignments were performed using computer software (Maven,18 Princeton, NJ), upon conversion of raw files into an.mzXML format using MassMatrix (Cleveland, OH).

### Data elaboration and statistical analysis

Replicates were exported as.mzXML files and processed through MAVEN.5.2; mass spectrometry chromatograms were created for peak alignment, matching and comparison of parent and fragment ions with tentative metabolite identification (within a 2-p.p.m. mass-deviation range between the observed and expected results against an imported KEGG database). To further explore the metabolic differences between the two groups of subjects, multivariate statistical analyses were employed on an MS data set consisting of 15 control subjects and 15 hypogonadal men. Multivariate statistical analyses were performed on the entire metabolomics data set using MetaboAnalyst 3.0 software, which also enabled an overview of the data variance structure in an unsupervised manner. Scatter plots were obtained using MetaboAnalyst 3.0. The web-based tools MSEA (Metabolite Set Enrichment Analysis) and MetPA (Metabolomic Pathway Analysis), which are incorporated into MetaboAnalyst platform, were used to perform metabolite enrichment and pathway analyses, respectively. For MSEA metabolites, data were mapped according to the HMDB, and the “metabolite pathway associated metabolites set” library (currently 88 entries) was chosen for the enrichment analysis which was performed using the package global test. Results were graphed with Graphpad Prism 5.01 (Graphpad SoftwareInc). Statistical analyses were performed with the same software. Data are presented as fold change ± SD.

## Results

### Metabolic profiling of plasma using HRMS

To explore the metabolic differences between the two participant groups, 15 control subjects and 15 IR hypogonadal patients were analysed, and multivariate statistical analyses were employed on the HPLC-MS data set. Untargeted metabolomic profiling of plasma from 2 independent cohorts of hypogonadal and control subjects was performed; 424 metabolites were detected across both groups and were further analysed using online tools (MetaboAnalyst 3.0 software). PCA revealed a clear separation of metabolomes between IR hypogonadal patients and control subjects. PCA was first used to investigate general interrelationships between the groups, including clustering and outliers among the samples (Supplemental Fig. 1).

**Fig. 1:**
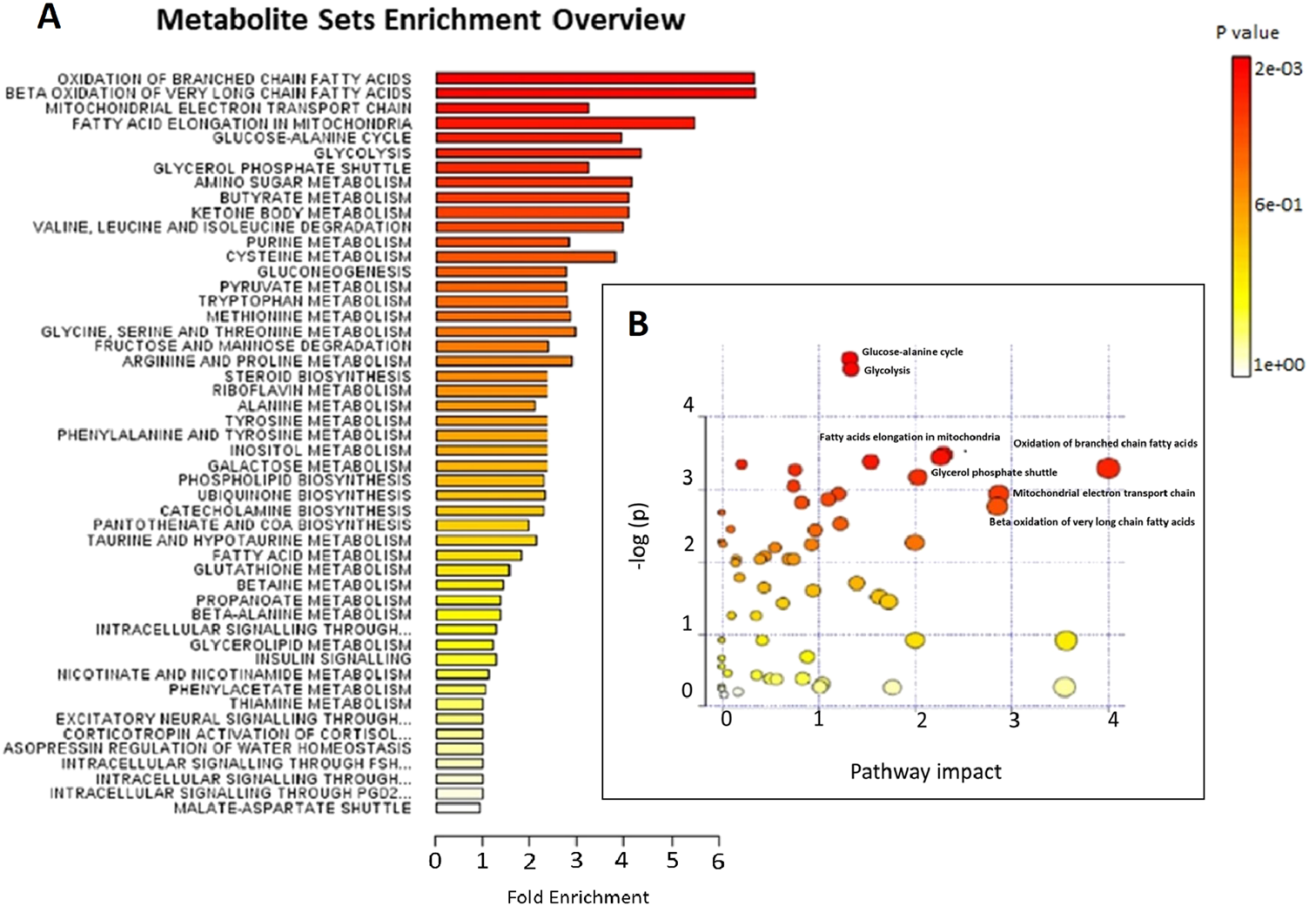
Metabolic Set Enrichment Analysis showing the most altered metabolisms as revealed in the plasma of hypogonadal men. Colour intensity (white-to-red) reflects increasing statistical significance, while the circle diameter covaries with pathway impact. The graph was obtained by plotting –log of p-values from pathway enrichment analysis on the *y*-axis the and the pathway impact values derived from pathway topology analysis on the *x*-axis. (**a**) Metabolic Pathway Analysis (MetPA). All the matched pathways are displayed as circles. The colour and size of each circle are based on the *p*-value and pathway impact value, respectively. The graph was obtained by plotting on the *y*-axis the −log of *p* values from the pathway enrichment analysis and on the x-axis the pathway impact values derived from the pathway topology analysis (**b**)

To identify which metabolic pathways were mostly affected in IR male hypogonadism, we performed an overview of pathways analysis according to *p*-values from their enrichment and impact values. The “metabolome view”, which shows all metabolic pathways, was arranged according to the scores from the enrichment analysis (*y*-axis) and topology analysis (*x*-axis) with the most significant *p*-values indicated in red and the least significant p-values in yellow and white. To understand the biological meaning of the observed metabolic changes, we made a functional enrichment analysis of the experimental data with MetaboAnalyst 3.0, which performs metabolite set enrichment analysis (MSEA) for human and mammalian species. The analysis is based on several libraries containing ∼6,300 groups of biologically meaningful metabolites at sets collected primarily from human studies. Figure. 1a, b represents the results of the pathway enrichment analysis conducted by MetaboAnalyst 3.0. Glycolysis is the main pathway affected, along with the Krebs cycle, as it is well-known that glucose is not used as the primary energy source in hypogonadism, especially in muscles. Interestingly, through MataboAnalyst 3.0 (Fig. 1a, b) it clearly appears that in insulin-resistant hypogonadism lipid metabolism represents the main alternative energy source to glucose pathways. In particular, elongation of fatty acid instead of their beta-oxidation is among the most altered metabolism, as well as oxidation of very long (VLCFFA) and branched (BFFA) fatty acid play a role is still under investigation.

### Glucose metabolism

**Fig. 2:**
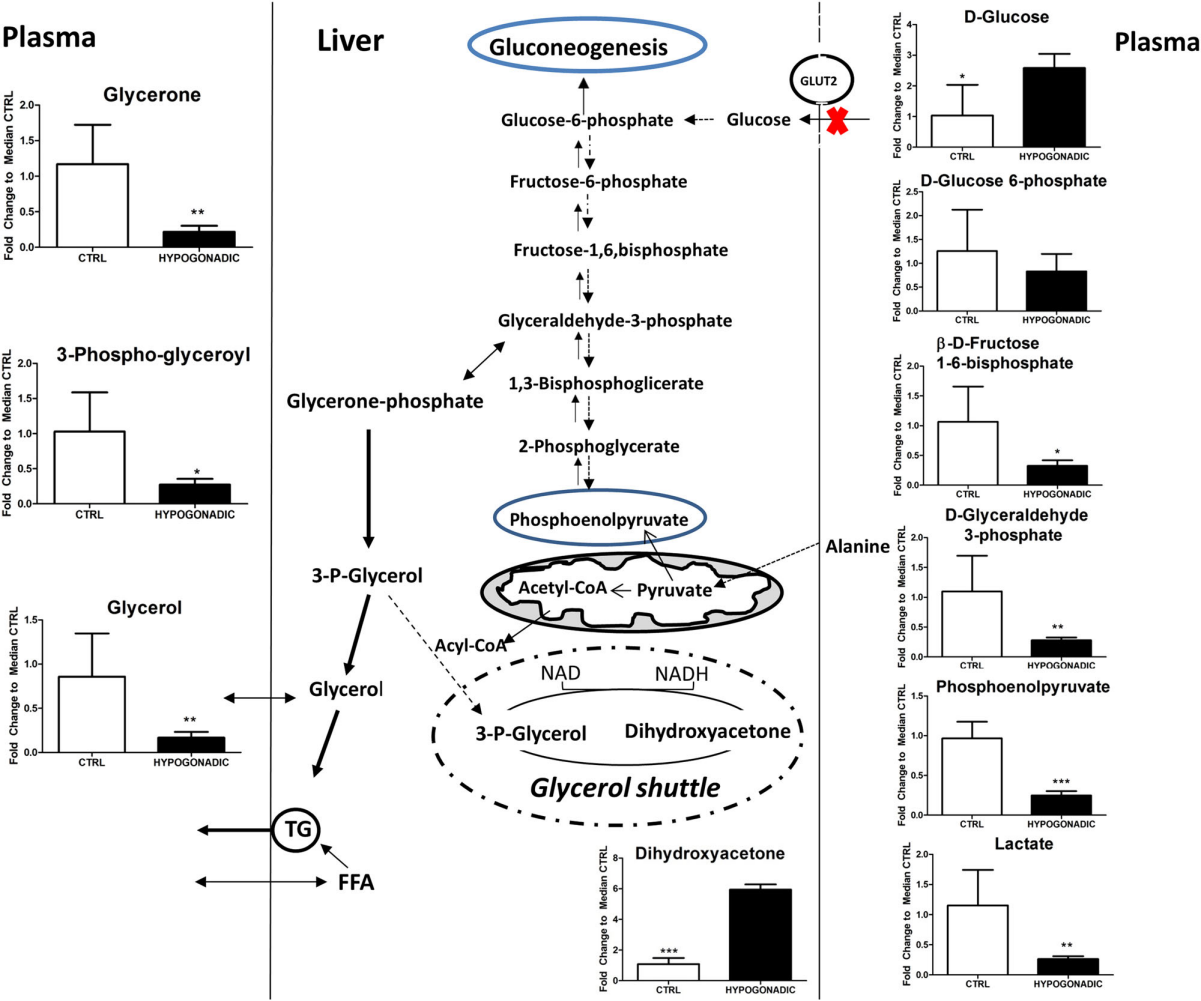
Intermediates of glycolysis and glycerol shuttling represented as the fold change of differences from control subjects vs hypogonadal patients. The total amount of glycolytic metabolites in the plasma appears to be reduced with respect to the levels in the control subjects. The columns present are expressed as the mean ± SD (*n* = 15) of the fold change in the metabolite concentration over hypogonadal plasma. *p < 0.05, **p < 0.01 ***p < 0.001 against hypogonadal men

In high insulin-resistant hypogonadism, GLUT4 expression is reduced in both muscle and adipose tissue^14,15^. As a result, glucose accumulates in plasma of these patients, reaching values 2.5-times higher (Fig. 2) than those in the control and IS hypogonadism groups^13^. Moreover, in liver, where glucose uptake could occur via a different membrane transporter (GLUT2), it has been demonstrated that testosterone has also a stimulatory effect on GLUT2 expression and glycogen phosphorylase activity^16–18^. Therefore, glucose can be produced through gluconeogenesis, a last resource alternative to obtain energy. Interestingly, in IR hypogonadism, gluconeogenesis is fuelled by the conversion of amino acids into glycolytic precursors. Consequently, in IR hypogonadism, the concentrations of glycolytic metabolites in plasma appear to be lower than in control subjects (Fig. 2). Phosphoenolpyruvate is an intermediate of both gluconeogenesis and lipid metabolism and is less reduced than other metabolites of glycolysis because it can be derived from amino acids. Moreover, only about 30% of the glyceraldehyde-3-phosphate is used for gluconeogenesis, while most of it being transformed into glycerone and then into glycerol (Fig. 2), for the synthesis of triglycerides. In fact triglycerides are higher in IR patients than in IS patients, suggesting that insulin plays a strong role in modulating lipogenesis (Table 1). Consequently, glycerol-3-phosphate does not participate in glycerol shuttling (Fig. 2), as indicated by the accumulation of dihydroxyacetone in IR hypogonadism with a parallel lower NADH production. The level of pyruvate, the end product of glycolysis, is strongly reduced in IR hypogonadism.

**Fig. 3:**
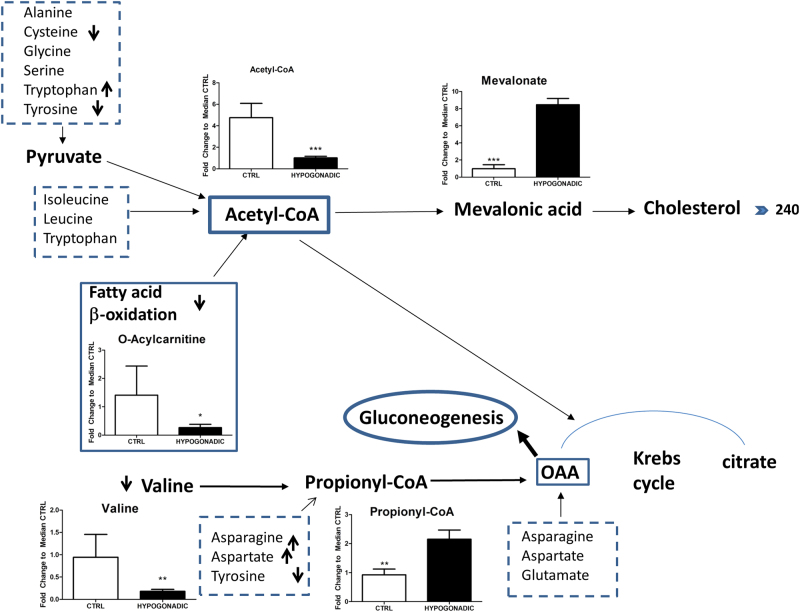
Metabolisms involved in Acetyl-CoA consumption or production as the main metabolite in glycolytic altered metabolism. Acetyl-CoA levels are strongly decreased in the plasma of hypogonadic men. Metabolites measured in the plasma are represented as the fold change of differences from control subjects vs hypogonadal patients. OAA is significantly re-increased as it is a precursor of gluconeogenesis produced from valine, asparagine, aspartate or tyrosine via fumarate. The columns present are expressed as the mean ± SD (*n* = 15) of the fold change in the metabolite concentration over hypogonadal plasma. *p < 0.05, **p < 0.01, ***p < 0.001 against hypogonadism

**Fig. 4:**
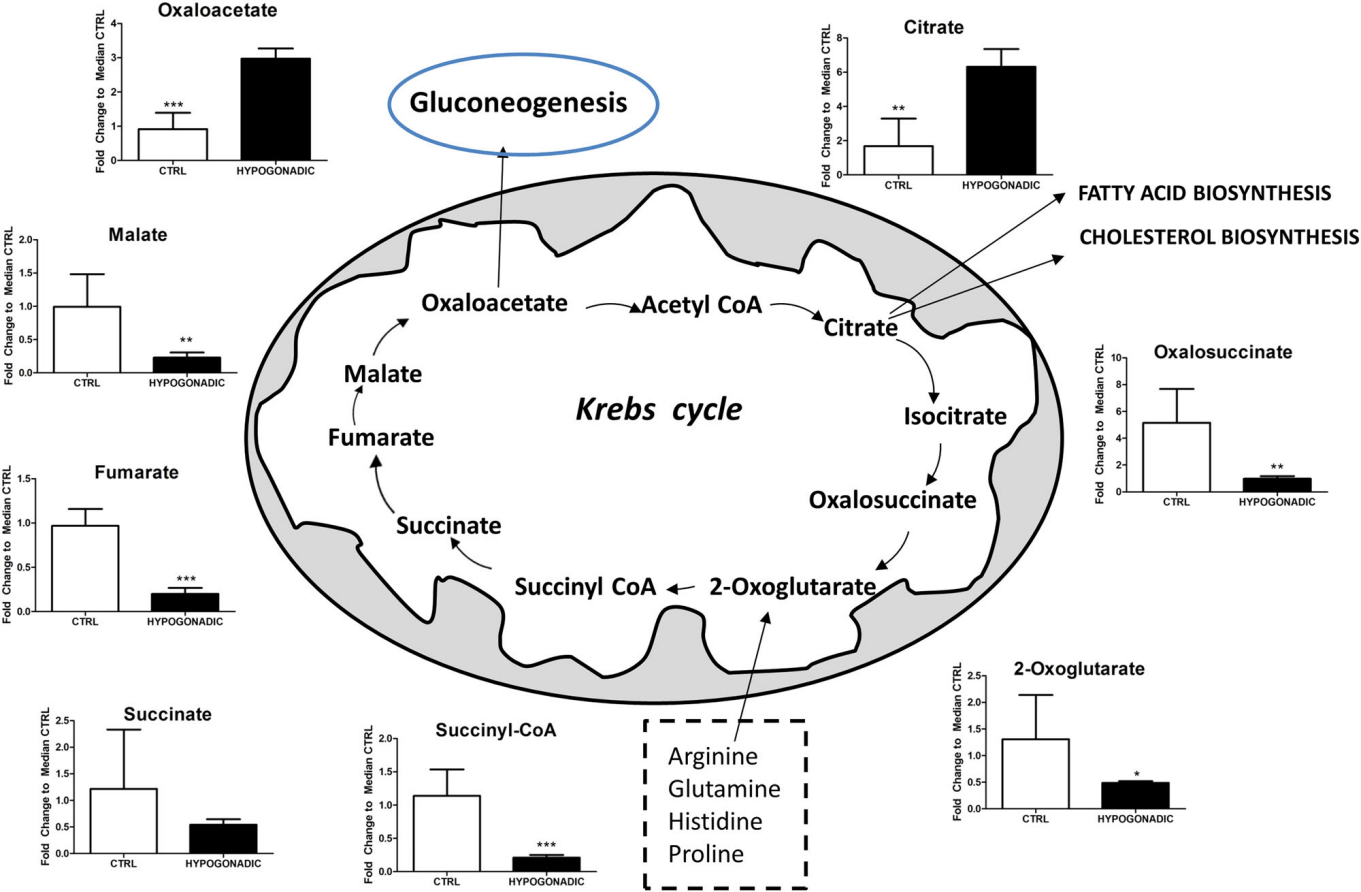
Intermediates of TCA measured in the plasma of hypogonadal patients, revealing that this metabolic pathway was strongly reduced. Metabolite levels are expressed as the mean ± SD (*n* = 15) of the fold change in the metabolite concentration over hypogonadal plasma. *p < 0.05, **p < 0.01, ***p < 0.001 against hypogonadal men

Acetyl-CoA levels are also strongly decreased (80%) in plasma of men with IR hypogonadism, even more than in subjects with IS hypogonadism^13^. Furthermore, although acetyl-CoA is the final product of beta-oxidation of fatty acids in healthy men, in IR hypogonadism, the beta-oxidation of fatty acids does not occur properly as shown by the acyl-carnitine levels (Fig. 3). Thus, acetyl-CoA appears to derive from some amino acids directly (Fig. 4) or indirectly through pyruvate.

In IR male hypogonadism, more acetyl-CoA is transformed into cholesterol, which increases up to 243 mg/dL (Table 1 and Fig. 3) as does its precursor mevalonic acid.

Finally, in IR hypogonadism, only a small percentage of acetyl-CoA enters the Krebs cycle, being transformed into citrate. Figure. 4 shows, through the intermediate products of the Krebs cycle, that the activity of this cycle was strongly reduced.

**Fig. 5:**
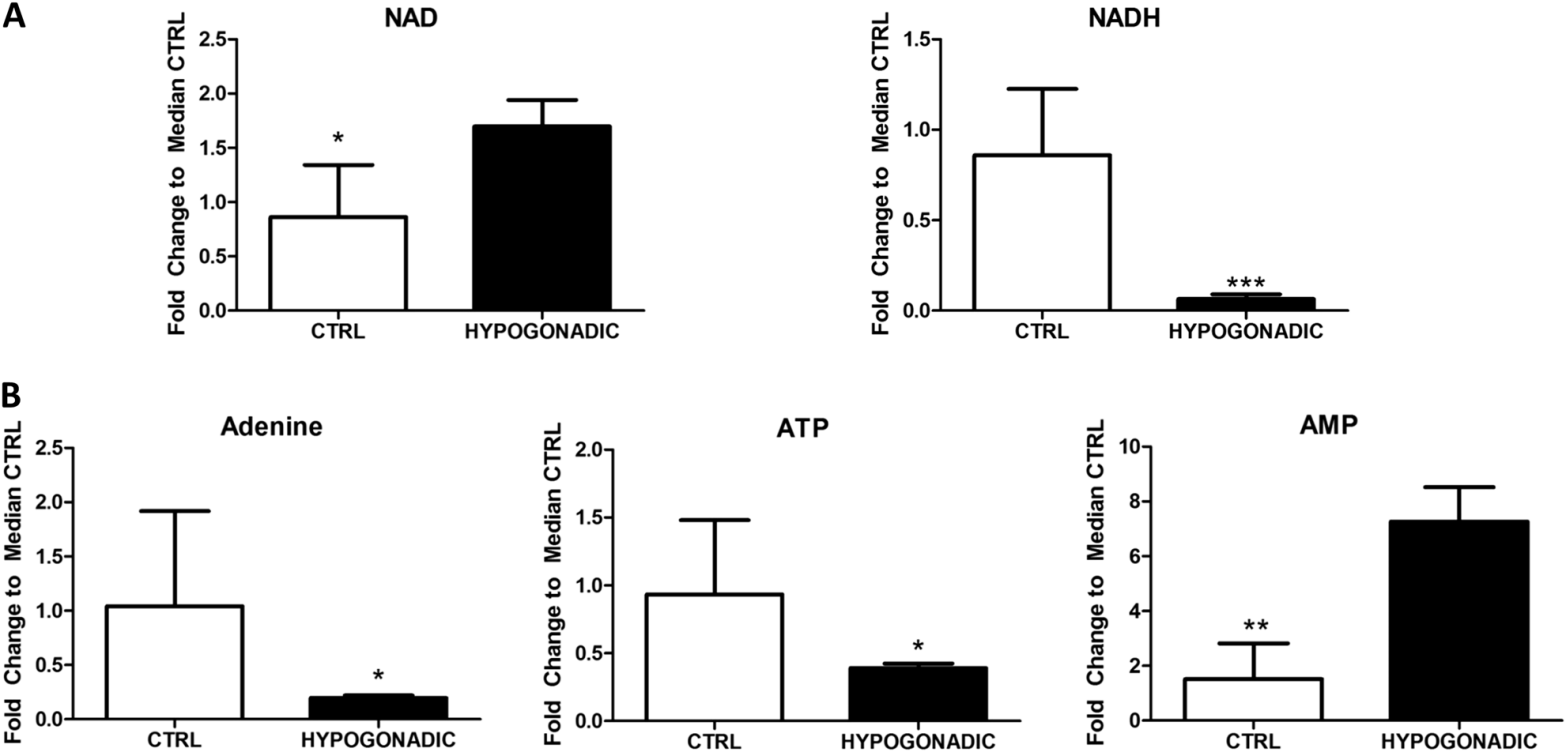
NAD, NADH, AMP and ATP changes in hypogonadism. Reduced Krebs cycle activity and glycerol shuttling induced reduced production of NADH (**a**) in hypogonadism, as well as that of ATP (**b**), both of which were paralleled by increase concentrations of NAD and AMP. Metabolite levels are expressed as the mean ± SD (*n* = 15) of the fold change in the metabolite concentration over hypogonadal plasma. *p < 0.05, **p < 0.01 ***p < 0.001 against hypogonadal men

**Fig. 6:**
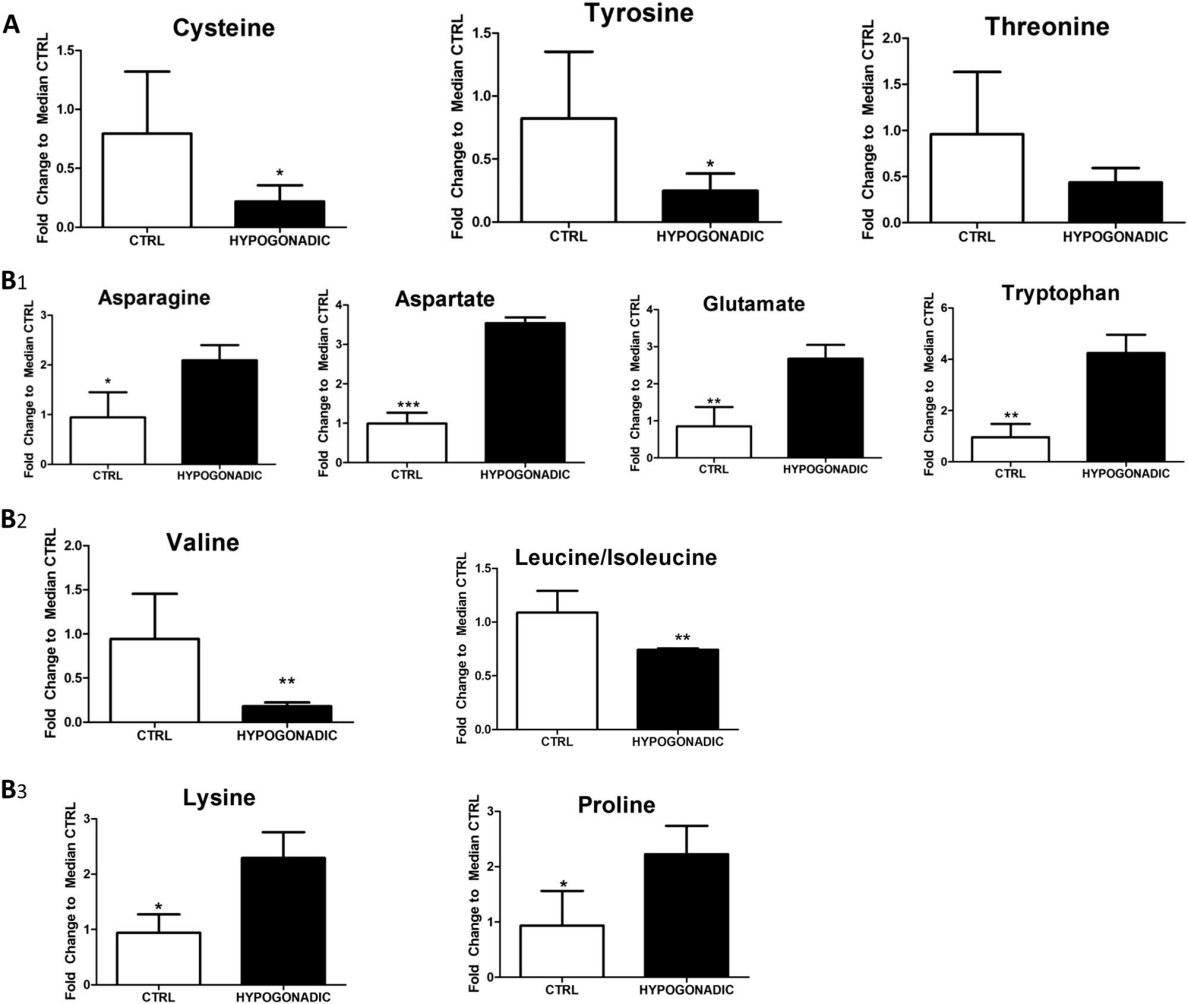
In insulin-resistant hypogonadism, some plasma amino acids were significantly reduced, while others were increased. Amino acids are represented as fold change of the differences from control subjects vs. hypogonadal patients. (**a**) Plasma amino acids reduced. (**b1**) Plasma amino acids that were significantly increased. (**b2**) Branched-chain amino acids (BCAAs): leucine, isoleucine and valine. (**b3**) Proline and lysine: amino acids that participate to form collagen fibres

Interestingly, the levels of citrate increased (by 70%) whereas those of oxalosuccinate decreased (by 80%), indicating that citrate is mostly consumed for the synthesis of fatty acids and cholesterol than for the Krebs cycle (Fig. 4). Besides citrate also OAA, a potential precursor of gluconeogenesis is significantly elevated being produced from valine, asparagine, aspartate or tyrosine (Fig. 3). For instance valine is strongly metabolised inside the mitochondria (approximately by 80%) to produce propionyl-CoA, which is finally transformed into OAA (Fig. 3). Consequently, the lower Krebs cycle activity and the reduced glycerol shuttle strongly reduced the production of NADH in IR hypogonadism, paralleled by an increase in NAD^+^ concentration (Fig. 5a). The reduction of NADH is also linked to altered mitochondria electron transport chain activity. Thus, altered oxidative phosphorylation was observed as predicted by MetaboAnalyst in Fig. 1a, which justifies a reduction in ATP production (by 60%) and an increase in AMP (by 80%), as shown in Fig. 6b.

### Amino acid metabolism

In insulin-resistant hypogonadism, amino acids play a main role. Some were found to be significantly reduced in plasma while others were increased. However, most of the non-polar amino acid levels (Fig. 6a, b) remained similar to those in the control group (Supplemental Fig. 2). The most affected amino acid metabolism pathways were those regarding cysteine, tyrosine, tryptophan, threonine, asparagine, proline and branched amino acids (valine and leucine/isoleucine). All these amino acids were significantly depleted in plasma. Conversely, significant increases were recorded for D-glutamate (70%). asparagine (55%), and aspartate (70%). The last amino acid gives rise to the production of OAA, a crucial Krebs’ intermediate for gluconeogenesis (Fig. 6B1).

Particular attention should be paid to the strong decrease of branched-chain amino acids (BCAAs): leucine/isoleucine and valine (Fig. 6B2).

Finally, among amino acids significantly increased, there were proline (40%) and lysine (70%) (Fig. 6B3), well known to participate in the formation of collagen fibres. Thus their higher level in hypogonadism is an indication of a reduction of collagen synthesis.

**Fig. 7:**
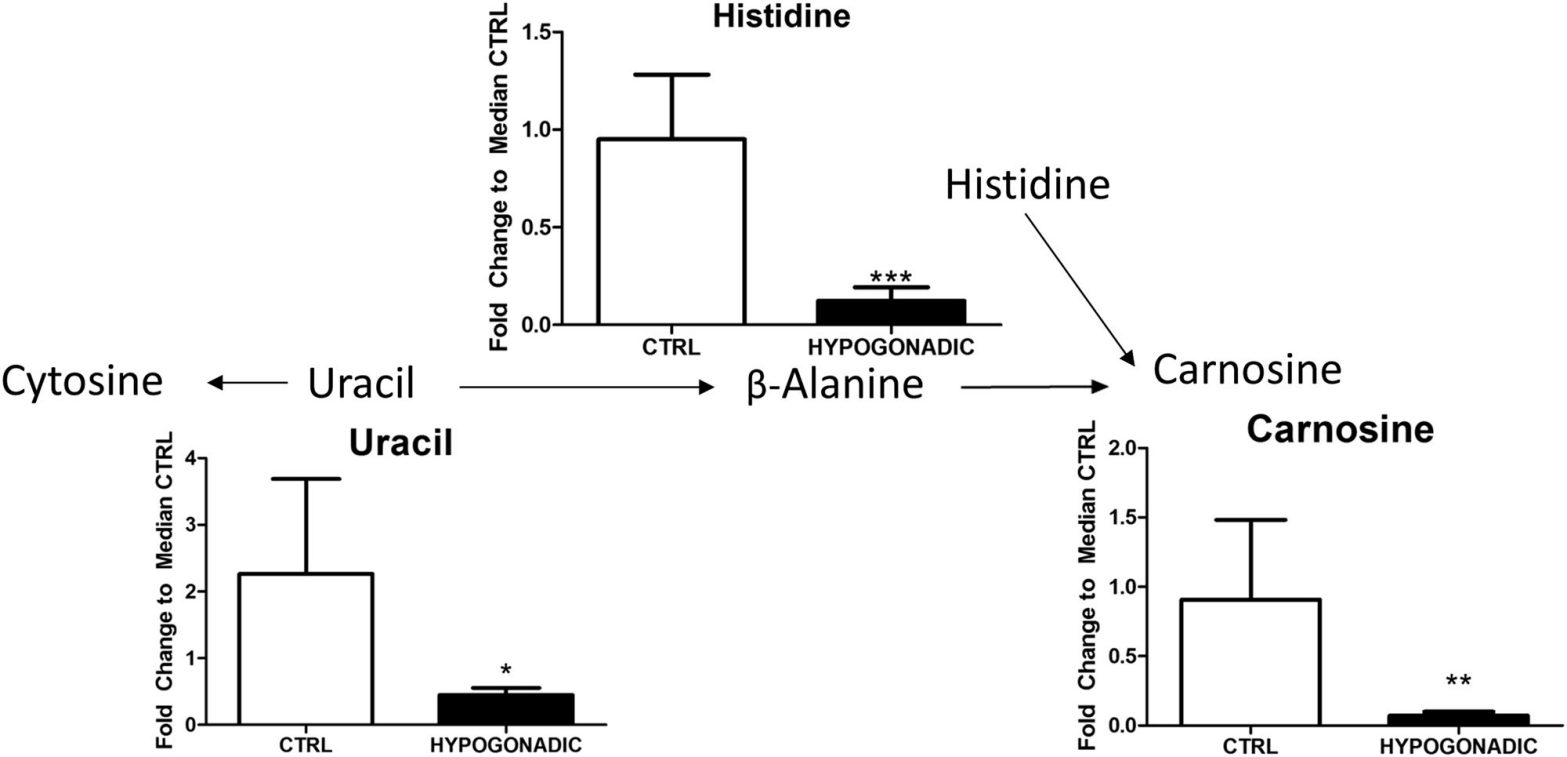
Metabolism of the production of carnosine from β-alanine. The metabolites are represented as the fold change of differences from control subjects vs hypogonadal patients. The columns present are expressed as the mean ± SD (*n* = 15) of the fold change in the metabolite concentration over hypogonadal plasma. *p < 0.05, **p < 0.01 ***p < 0.001 against hypogonadal men

In hypogonadism, carnosine, as well as its component β-alanine and uracil, from which the latter is derived are strongly decreased (Fig. 7) as already predicted by MetaboAnalyst (Fig. 1a).

Of note is the abnormal increase of betaine in IR hypogonadism (Supplementary Fig. 3).

## Discussion

In IR hypogonadism, our HRMS metabolomics analysis revealed that approximately 38 canonical biochemical pathways were affected, among which 18–20 pathways (red bars) in a very significant way.

### Glycolytic pathways

Glycolysis is the most consistently altered biochemical process. Most likely, this is a consequence of a significantly reduced GLUT4 expression in muscle and adipose tissue in men with IR hypogonadism independent of circulating insulin levels^19^. Furthermore testosterone has also a stimulatory effect on GLUT2 expression and glycogen phosphorylase activity^15^. Therefore, it is not surprising that increased glucose detected in plasma and liver of hypogonadic men deriving from gluconeogenesis is the last resource for obtaining glucose. Surprisingly, lactate levels were abnormally low in men with IR hypogonadism with respect to IS hypogonadism^13^, most probably because in these patients, lactate enters to liver to fuel gluconeogenesis. Consequently, since there is a correlation between lactate and testosterone production in rat Leydig cells^20^, in these patients, testosterone production can be further reduced upon time.

Our metabolomics analysis revealed that the Krebs cycle is significantly hindered at the citrate-isocitrate level, which is used for lipogenesis in IR; thus, less energy is produced through the Krebs cycle. In fact reduced ATP levels in conjunction with an increase of AMP in the plasma, were observed. This is in agreement with Pitteloud et al^11^. who reported an inverse correlation between testosterone levels and impaired mitochondrial function. In IR patients, the presence of insulin resistance of muscle cells augmented mitochondrial capacity and fostered the expression of genes relating to oxidative phosphorylation^21^.

### Lipid pathways

Fatty acids are not efficiently burned by β-oxidation in hypogonadism, as indicated by the lower acetyl-carnitine level, which is fundamental for fatty acids transport into the mitochondria (Fig. 2). It should be noted that acetyl-carnitine level in plasma is a good biomarker of β-oxidation of fatty acids^22^. Therefore, β-oxidation of common fatty acids did not represent a significant energy source in hypogonadism, at variance with long and branched fatty acids, as predicted with MetaboAnalyst 3.0 (Fig. 1a).

Interestingly, 3-phosphoglycerolphosphate (G3P) does not participate in glycerol shuttle, which is strongly reduced, because G3P is mainly used to produce triglycerides (TGs). As a matter of fact, when mammals cannot use carbohydrates to generate ATP, glucose is mostly converted into fatty acids (lipogenesis) for synthesis and storage of TG in liver and white adipose tissue^23^. A higher free fatty acids concentration was indeed recorded^21,24^, which shifts glycerol consumption toward triglyceride formation. Thus, the liver of males with IR hypogonadism is more prone to lipogenesis than that in males with IS hypogonadism; therefore the higher the insulin levels, the stronger is the stimulation of lipogenesis with increased triglycerides up to 211 mg/dL. In IR hypogonadism, obesity increases significantly, in a BMI range = 30.29 ± 4.43 (Table 1). Moreover, elevated production of triglycerides in non-adipose tissues, such as liver, induces the overexpression of lipoprotein lipase and contributes to insulin resistance^23^. Insulin promotes glucose uptake and regulates triglyceride catabolism through the inhibition of hormone-sensitive lipase^23^.

It is of note that plasma cholesterol is approximately similar to the levels recorded in IS hypogonadism (ranging between 213 to 243 mg/dL) indicating that insulin does not significantly influence this metabolism in IR hypogonadism.

### Amino acid metabolism

Our analysis revealed that pyruvate was not efficiently produced through glycolysis, but rather from the reactions involving glutamate and alanine. Thus, glutamate accumulation in the liver stimulates gluconeogenesis and contributes to the development of glucose intolerance, as described by Newgard et al^25^. for obese subjects.

As observed in IS hypogonadism^13^, IR hypogonadism is associated with increased proline and lysine levels. Since these two amino acids are involved in collagen synthesis, it may be hypothesised that their accumulation in plasma is an indication of defective bone formation. This could explain the osteoporosis commonly observed in individuals with hypogonadism^26,27^. Our analyses suggest that osteoporosis is related to testosterone deficiency^28–30^ and is independent from insulin activity.

Three specific amino acids, leucine/isoleucine and valine, play a role in IR hypogonadism. They account for nearly 35% of the essential amino acids in muscle proteins and approximately 40% of the essential amino acids required for mammal’s diet^31^. The higher protein catabolism to produce energy causes a decrease in muscle mass. A gain in fat and lose in muscle is typical of hypogonadal patients. Recent studies showed that metabolome profiling of obese versus lean humans revealed a BCAA-related metabolite signature that is suggestive of increased catabolism of these amino acids and is correlated with insulin resistance^25,32^. In a dietary regime that includes high fat consumption, BCAAs contribute to the development of obesity-associated IR, and plasma shows increased BCAA levels^25,32^. In contrast, in case of testosterone deficiency, BCAA levels are decreased because they are oxidised to supply energy. In agreement with this, it was determined that subjects considered obese had higher metabolic rates of BCAAs and increased resistance to insulin than lean individuals with a lower body mass index.

### Other affected pathways

Beside the reduction of muscle mass in IR hypogonadism, a slower degradation of uracil produced less β-alanine, a precursor of carnosine, leading to a reduced muscle activity. This agrees with evidence that upon orchiectomy, carnosine levels significantly decrease in male mice^33^, which can be restored by testosterone replacement, suggesting a relationship between testosterone and carnosine concentration. Carnosine levels in muscle contribute to better performances of high-intensity exercise^34^. Thus, it is not surprising that β-alanine is a popular supplement used primarily by strength and power athletes to enhance their performance, as well as for training aimed at improving muscle growth, strength and power. In hypogonadism, the reduced muscle mass associated to reduced carnosine levels can explain the sense of fatigue and mental confusion^35^ commonly observed in these patients.

Interestingly, in IR hypogonadism is observed an increased catabolism prevailing on anabolism, thus leading to increased osmotic stress in tissue (especially liver). Therefore betaine levels increase enormously because of its role as an osmo-protector. An increase of betaine was also observed in altered metabolism and contributes to counteract hepatic, vascular, coronary, and cerebral diseases^36^. Betaine levels are increased in liver diseases characterised by the development of fatty liver, especially non-alcoholic fatty liver disease^37^.

**Fig. 8:**
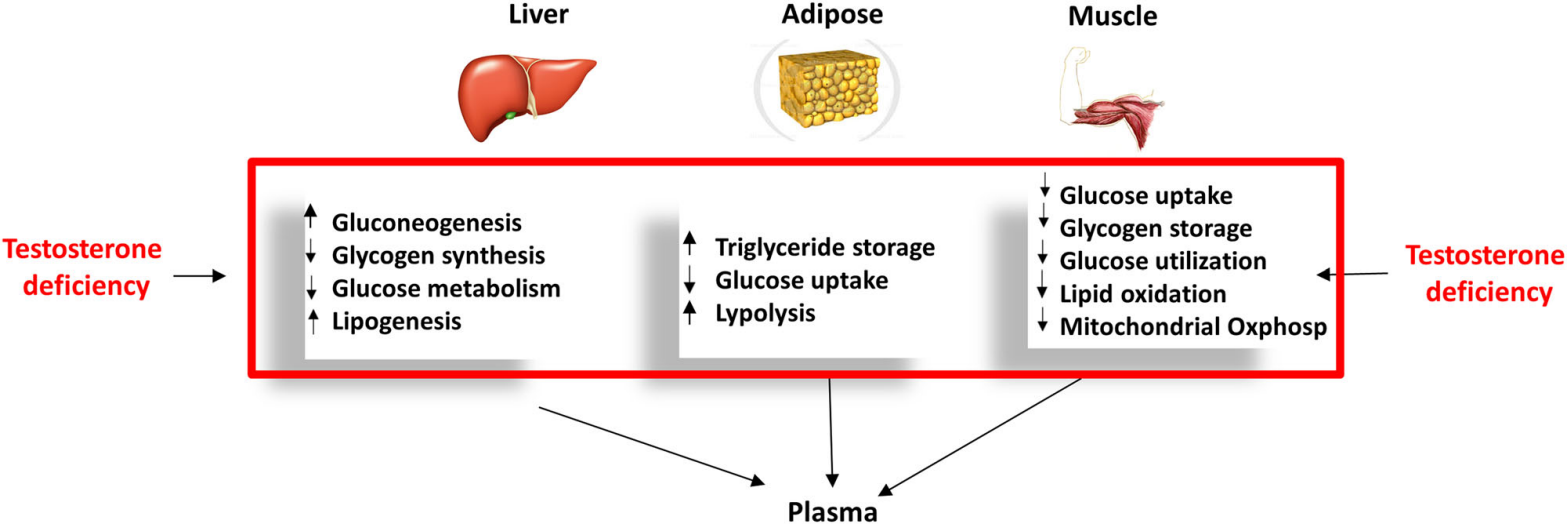
Schematic representation of the metabolisms affected by testosterone deficiency in the three main tissues affected by hypogonadism. Boxes report metabolisms notoriously altered by testosterone deficiency (increased or decreased). This figure also shows that plasma from a male with hypogonadism is the biofluid in which the metabolites are passively excreted from all other tissues

In conclusion, the analysis of plasma metabolites appears to be a valid way toward a better diagnosis of hypogonadism and catalogue the individuals into sub-categories based on specific metabolic alterations rather than general symptoms. We conducted for the first time an exhaustive and simultaneous assessment of all metabolisms and simplified the correlation among altered glucose, protein and lipid metabolism. Figure. 8 summarises the mainly involved tissues, with the metabolic pathways most affected in IR male hypogonadism in agreement with a recent review^14^. This work underlines the importance of using a systems biology approach for future research. In fact elucidating metabolic pathway changes in hypogonadism allows a better understanding of the mechanism of “metabolic syndrome” correlated with low levels of testosterone and its relationship with insulin resistance. This knowledge could help to predict the response to treatment and how to limit complications in untreated patients. Clinically, studying IR hypogonadism could help with the development of gluconeogenesis precursors, as well as integration of amino acids, especially leucine, isoleucine and valine, into treatments. Carnosine and β-alanine also should be supplemented. This could help improve testosterone therapy, which does not completely restore hypogonadic metabolisms.

## Electronic supplementary material

Supplemental Figure 1

Supplemantal Figure 2

Supplemental Figure 3

Supplementary figure legends
