# Supplementary figures and images for: Metabolic patterns in insulin-resistant male hypogonadism
Source: Cell Death Dis. 2018 Apr 22;9(6):671. doi: 10.1038/s41419-018-0587-9 (PMC5986816; doi:10.1038/s41419-018-0587-9)

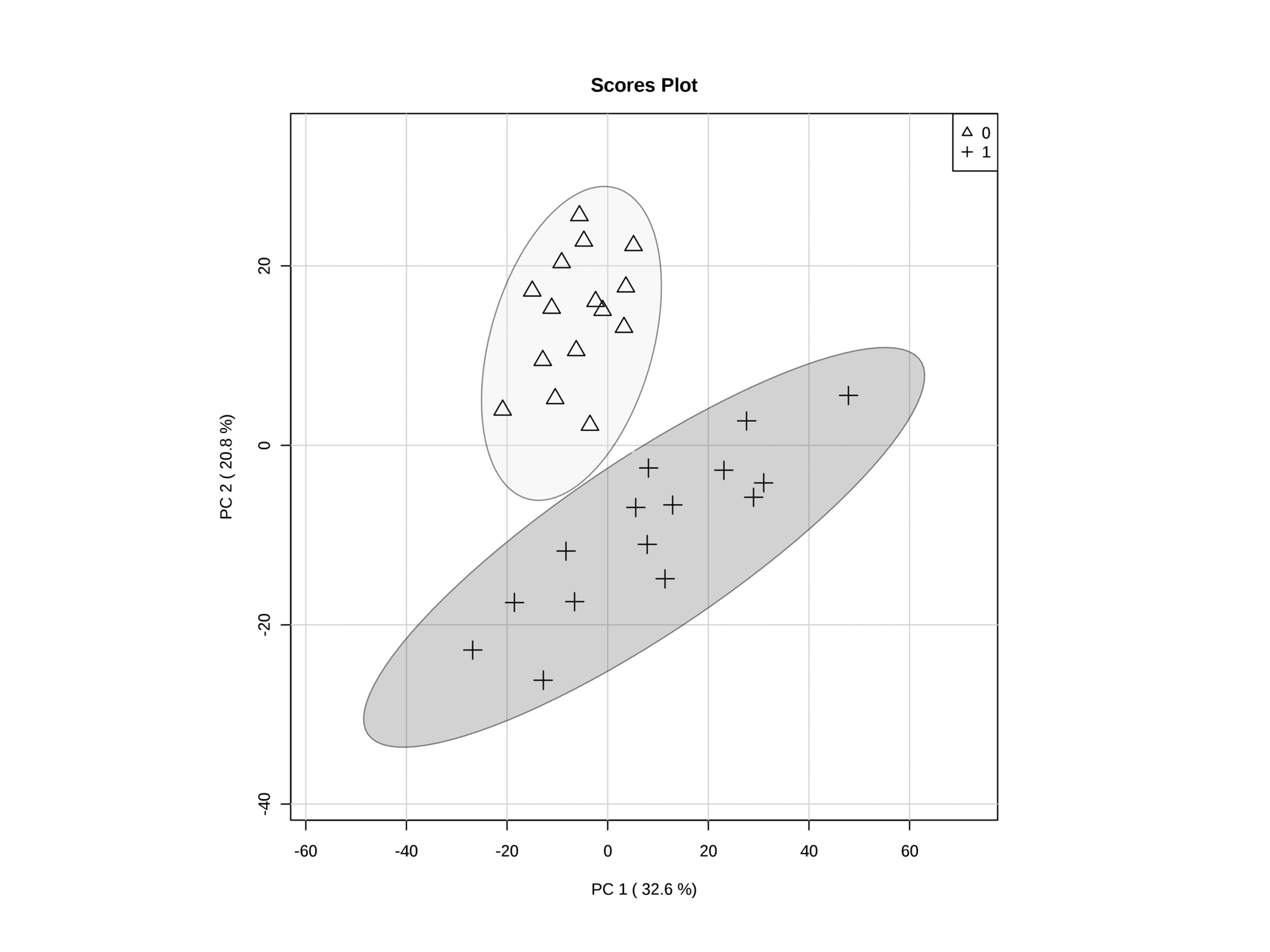

Supplement: Supplementary file 1 — Supplemental Figure 1 [file 41419_2018_587_MOESM1_ESM.tif]

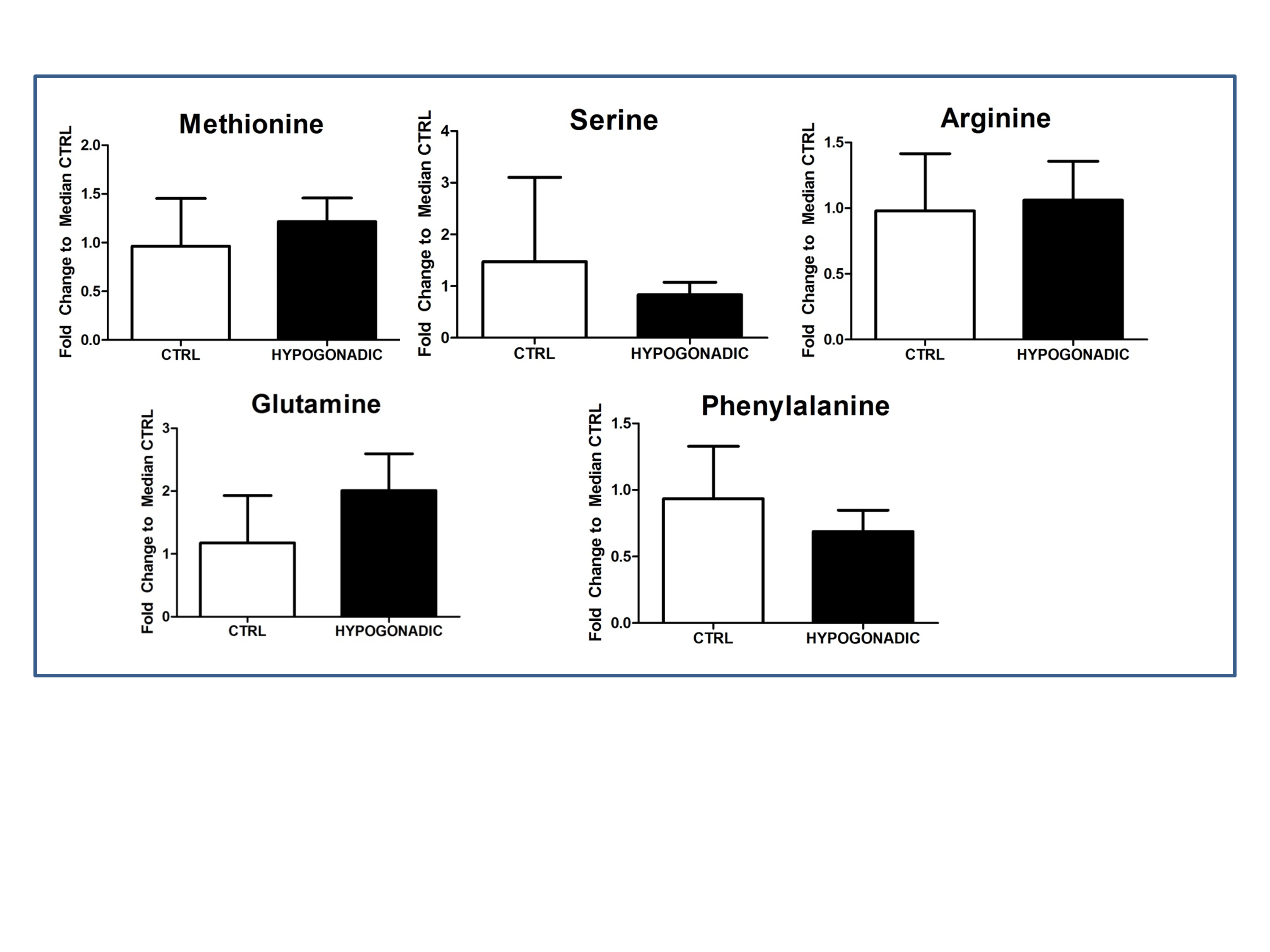

Supplement: Supplementary file 2 — Supplemantal Figure 2 [file 41419_2018_587_MOESM2_ESM.tif]

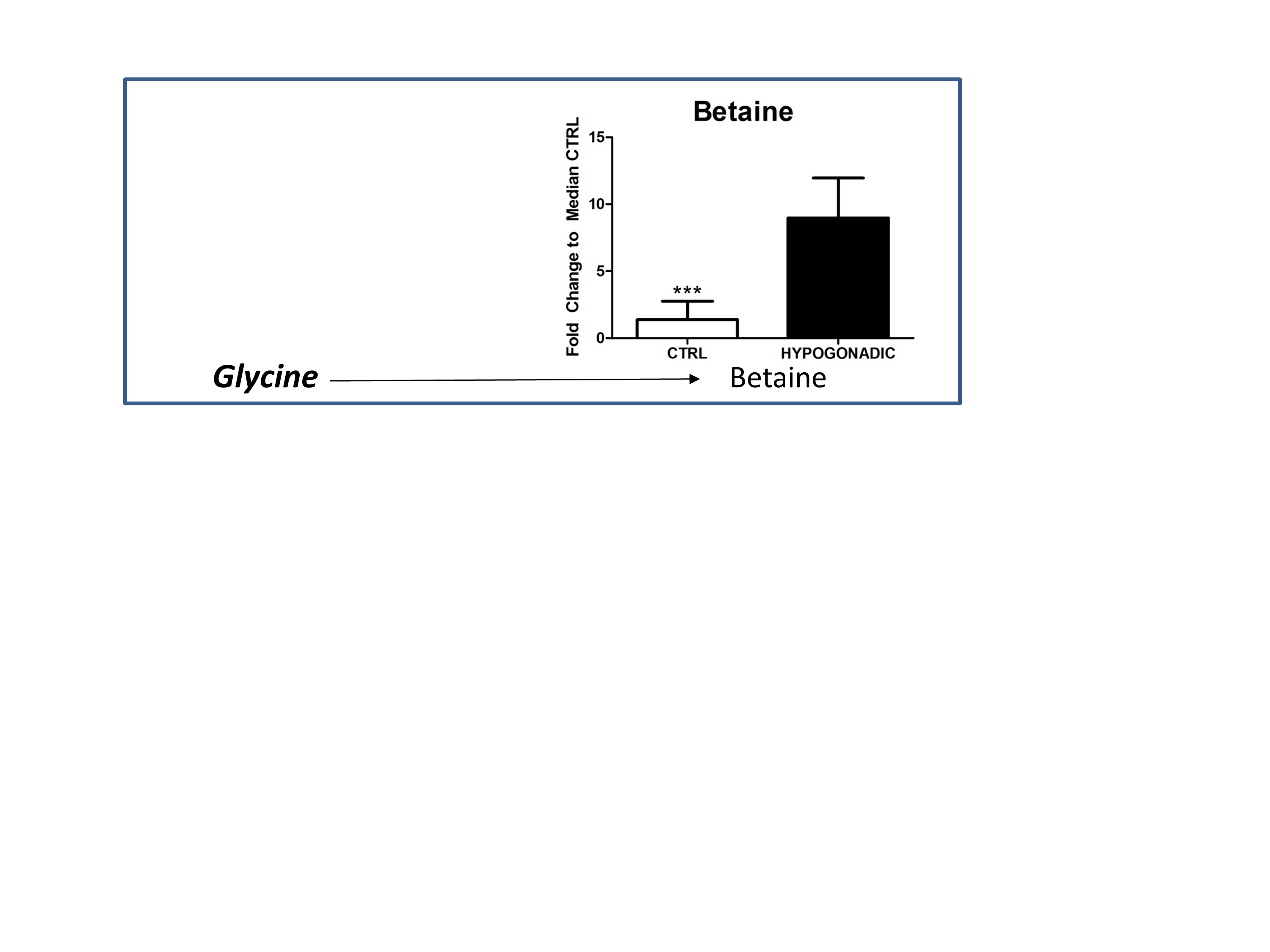

Supplement: Supplementary file 3 — Supplemental Figure 3 [file 41419_2018_587_MOESM3_ESM.tif]
