# Supplementary material for: Metabolic patterns in insulin-resistant male hypogonadism
Source: Cell Death Dis. 2018 Apr 22;9(6):671. doi: 10.1038/s41419-018-0587-9 (PMC5986816; doi:10.1038/s41419-018-0587-9)
Supplement: Supplementary file 4 — Supplementary figure legends [file 41419_2018_587_MOESM4_ESM.docx]

**Supplemental Figure 1**: Multivariate model generated from the HRMS analysis of human plasma. PCA analysis shows separation of control subjects (grey light) and hypogonadic patients (grey dark).

**Supplemental Figure 2**: Amino acids that remain similar to between control subjects and hypogonadic patients. The columns present are expressed as the mean ± SD (n = 15) of the fold change in the metabolite concentration over hypogonadal plasma. *p < 0.05, **p < 0.01 ***p < 0.001 against hypogonadal men.

**Supplemental Figure 3**: Production of betaine from glycine. The columns present are expressed as the mean ± SD (n = 15) of the fold change in the metabolite concentration over hypogonadal plasma. *p < 0.05, **p < 0.01 ***p < 0.001 against hypogonadal men.
